# Estrogen and Progesterone Expression in Colorectal Carcinoma: A Clinicopathological Study

**DOI:** 10.31557/APJCP.2020.21.4.1155

**Published:** 2020-04

**Authors:** Asmaa Abd ElGhany Abd ElLateef, Ahmed El Sayed Mohamed, Ahmed AS Elhakeem, Sheren FM Ahmed

**Affiliations:** 1 *Department of Clinical and Radiation Oncology, Faculty of Medicine, Sohag University, *; 2 *Department of Pathology, Faculty of Medicine, AL-Azhar University, Assiut, *; 3 *Department of Pathology, Faculty of Medicine, Sohag University, Faculty of Medicine Egypt. *

**Keywords:** ER, PR, Colorectal cancer, Immunohistochemistry

## Abstract

Sex steroids have been suggested to influence colorectal cancer (CRC) carcinogenesis. Also, exposure to exogenous hormones might contribute to its incidence. This study conducted to evaluate ER and PR expression as a prognostic factor in patients with CRC attending Sohag University Hospital (SUH) and Sohag Cancer Center (SCC). Materials and Methods: Tumor samples tested for Estrogen receptor (ER) / progesterone receptor (PR) expression using immunohistochemical staining (IHC). Association of this expression with overall survival (OS), disease-free survival (DFS) and progression-free survival (PFS) were evaluated. Results: Thirty out of 50 CRC tissues were evaluable for hormone receptor expression. Expression of both ER and PR was cytoplasmic. ER and PR expressions were 60% and 76.66%, respectively. There was a significant difference between loss of ER expression and depth of invasion (p= 0.01). Also, ER and PR negative expression cases were significantly at higher risk for progression (p= 0.03; 0.009 respectively). High levels of ER and PR expression were associated with higher cumulative PFS at one year and at the end of follow up time (p=0.01; 0..02 respectively); however this did not reach statistical significance on Cox proportional hazards regression analysis for progression or OS (p= 0.05; HR= 0.22; p=0.5; HR=0.67 respectively) for ER level and (p=0.07; HR=0.22; p=0.6; HR=0.72 respectively) for PR level. Conclusions: This study suggests that lower ER/PR expression levels were associated with more extensive CRC primary tumors and poorer prognosis. These data suggest that ER/PR expression might possess a prognostic value for CRC cases.

## Introduction

Colorectal cancer (CRC) is a major health problem worldwide with high mortality rates (Bray et al., 2018) and higher predominance in males than females (American Cancer Society, 2017). In Egypt, CRC is the 7^th ^commonest cancer; representing 3.47% of male cancers and 3% of female cancers (Ibrahim et al., 2014).

Steroid hormones have an established physiological role in reproductive system, bone, cardiovascular and brain functions (Burns and Korach, 2012; Ascenzi et al., 2006; Deroo and Korach, 2006). However, Estrogen also has a role in different pathological diseases as well as certain tumors. Estrogens have oncogenic and tumor promoter effect linked to different tissue types, breast (Jordan, 2007), ovary (Syed, 2001), uterus (Zannoni et al., 2013), and prostate (Härkönen and Mäkelä, 2004), lung (Siegfried, 2014) and colon (Hogan et al., 2009). ER modulators and drugs affecting ER biosynthesis are highly successful therapeutic agents for breast cancer patients (Hua et al., 2018).

Sex steroids have been suggested to influence CRC carcinogenesis. Supported by a number of clinical and laboratory observations; CRC incidence tends to be lower in females than in males suggested that ovarian steroids may be contributing factors. Also, oral contraception and hormonal replacement therapy are associated with reduced CRC risk (Stevanato Filho et al., 2018). Presence of estrogens was associated with lower risk of CRC as shown by In-vitro and epidemiological studies (Weyant et al., 2001; Foster, 2013) and The Women’s Health Initiative findings supported that postmenopausal women treated with hormone replacement therapy had lower colon cancer incidence (Rossouw et al., 2002).

Estrogen receptor beta (ERβ) is by far the predominant isoform in the colon mucosa (Campbell-Thompson et al., 2001; Konstantinopoulos et al., 2003; Wong et al., 2005) and its expression is lost during the progression of colon cancer (Foley et al., 2000; Wada-Hiraike et al., 2006). Also, Elevated ER beta expression was associated with a better prognosis in patients with CRC as shown in recent studies (Stevanato Filho et al., 2018, Topi et al., 2017). Also, the presence of both ER and progesterone (PR) expression was associated with lower proliferation and more apoptosis of colon cancer, probably through ER receptor beta activation (Sasso et al., 2019). 

Some studies reported loss of PR expression in colon tumors and lack of its carcinogenic effect in animal models (Heijmans et al., 2011). However, other studies reported that PR expression level has a significant difference between normal colon, adenoma and adenocarcinoma, supporting its role in this disease (Qasim et al., 2011). However, studies showing a prognostic role for ER and PR in CRC were with inconclusive results. This work conducted to evaluate the IHC expression of ER and/or PR as prognostic factors in patients with CRC.

## Materials and Methods

*Tissue samples*

50 CRC adenocarcinoma samples were collected; thirty of them were eligible to be included within this study as 20 fine needle biopsies failed to provide sufficient tissue for analysis. These samples collected from Sohag University Hospital (SUH) and Sohag Cancer Center (SCC). Twenty five were obtained from radical surgery while five from lower endoscopic biopsies. The clinical data of these cases were collected from their medical reports. 

*Immunohistochemistry*

The antibodies and chromogen detection system used in this work were purchased from Thermo Scientific. Four micrometer-thick sections from formalin-fixed paraffin-embedded tissue blocks of the tumor tissues were de-paraffinized in xylene and rehydrated in down-regulated alcohols. The sections were washed in running water before incubation in 0.5% hydrogen peroxide for 10 minutes to block the endogenous peroxidase activity and then washed in running water. Antigen unmasking was by boiling in 10 mM citrate buffer, pH 6.0 in a microwave at high power for 20 minutes. Following antigen retrieval, the sections were left to cool down for 30 minutes and washed in Tris-buffered saline (TBS) pH 7.6. Fifty micron of mouse monoclonal anti-human ER primary antibody (Thermo Scientific, clone 6A12, Richard Allan Scientific Co, USA) and rabbit monoclonal anti-human PR primary antibody (Thermo Scientific, clone RM-9102-S0, Richard Allan Scientific Co, USA) diluted in TBS with dilution of 1:100 for both and was put on each tissue section and the slides were incubated overnight at 4°C. Next day, the sections were washed in TBS before incubation with peroxidase-labelled goat anti-mouse secondary antibody for 10 minutes at room temperature. The sections were washed with 0.5% TBS and exposed to 3,3′- diaminobenzidine tetrahydrochloride (DAB) solution to yield an insoluble brown deposit. Finally, the sections were counterstained with hematoxylin, dehydrated and mounted as usual. Replacement of the primary antibodies with TBS served as negative controls for the immunohistochemistry (IHC) process. Positive control for ER and PR was considered from endometrial tissues (proliferative and secretory phase respectively).

*Scoring*

The IHC results were scored and analyzed. Slides were assessed by reviewing them at 40x and 100x magnification to assess the distribution and intensity of the stain and at 200x and 400x magnification to semi-quantitatively evaluate the scoring parameters of the immunostaining. The immunoreactive score (IRS) was determined by multiplying an estimate of the percentage of the immunoreactive cells with an estimate of the staining intensity as by Sasso (2019). Staining quantity is scored as follows: No staining= 0, <10% of cells stained= 1, 11-33% of cells stained = 2, 34-65% of cells stained = 3 and >65% of cells stained= 4. Staining intensity is scored on a scale of 0-3 where: no staining= 0, weak= 1, moderate= 2 and strong= 3. An IRS of 0 was considered negative, 1-4 was weak, 6 and 8 was moderate, 9 and 12 was considered strong (Fong et al., 2014).

*Statistical analysis*

Data was analyzed using STATA intercooled version 12.1. Qualitative data was presented as number and percentage and compared using Chi square test. The log-rank test was performed to evaluate significant differences between survival curves of different variables. Hazard ratios (HR) with 95% confidence intervals (CI) and P values were estimated with respect to the reference category for each co-variate. Graphs were produced by using STATA program. P value was considered significant if it was less than 0.05. 

## Results

**Figure 1:**
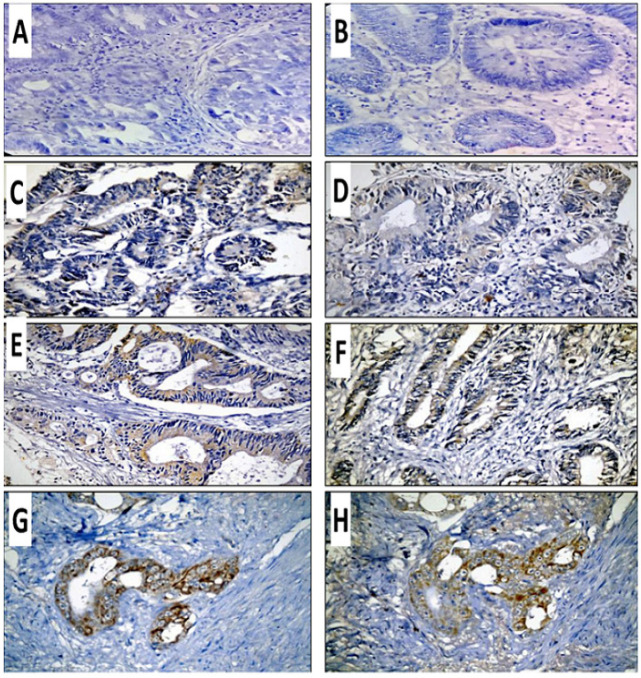
Expression of ER and PR in Cases of Colorectal Adenocarcinoma; Negative ER (A) and PR Expression (B), Weakly Expressed ER (C) and PR (D), Moderately Expressed ER (E) and PR(F), and Strong expression of ER (G) and PR (H). Original magnification is 400X

Thirty patients were included within this study. The median age was 55 years (range from 27-85); while the mean age was 53.07 (SD ± 14.19). Males were 53.3 % and 46.7% were females. The majority of patients had left-sided tumors (80 %). Grade III tumors found in 60% of cases. More than two thirds (70 %) had advanced stage and nearly one third presented by metastatic disease. More than two thirds failed frontline treatment. The median PFS for total cohort was 9 (4-53) while the median OS was 27.5 (2.4-58.7). Expression of ER and PR was cytoplasmic with combined cytoplasmic and focal nuclear expression in only two cases in < 10% of positive cells (Figure 1) and ER and PR expression rate was 60% and 76%, respectively (Table 1).

Comparative analysis showed significant association between loss of ER expression and greater tumor extension (p= 0.01). Also, ER expression negativity on tumor specimen was significantly associated with worse clinical outcome; patients are more likely to progress clinically if their tumor has no ER expression (p= 0.03). Also, PR positive cases showed significant likelihood for better clinical outcome (p= 0.009) (Table 2).

**Figure 2:**
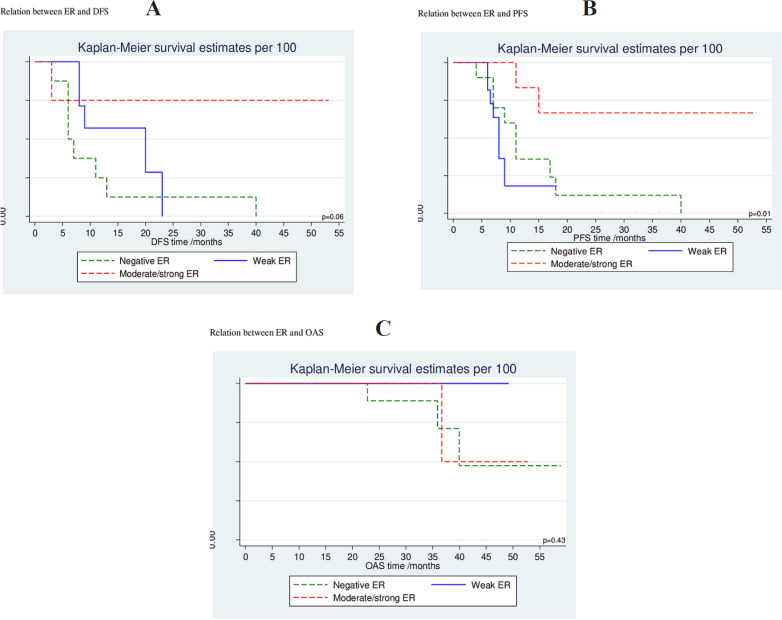
Association of ER Expression with DFS (A), PFS (B) and OAS (C) of the Studied Cases

**Figure 3:**
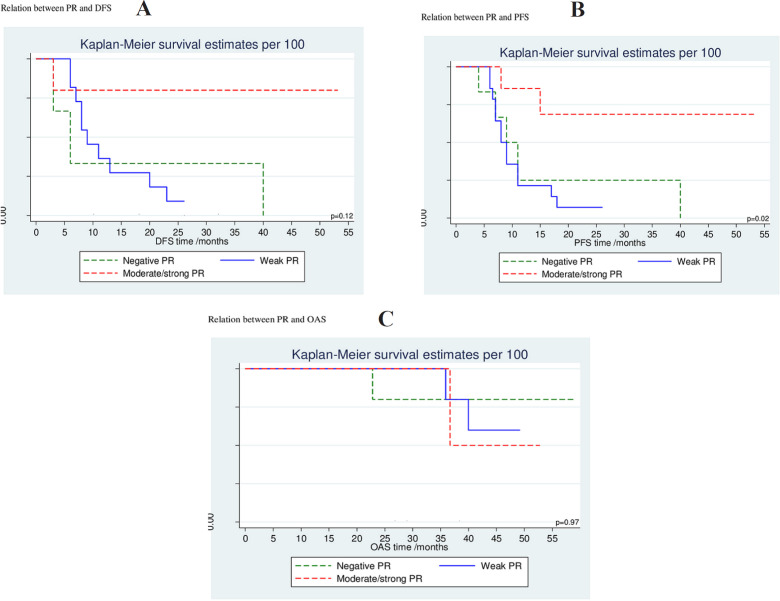
Association of PR Expression with DFS (A), PFS (B) and OAS (C) of the Studied Cases

ER negativity was significantly associated with higher cumulative probability of more progressive disease while moderate and/or strong ER expression was associated with lower progressive disease with 66 % cumulative PFS at 53 ms (p= 0.01); however, this did not reach statistical significance on Cox proportional hazards regression analysis for PFS (p= 0.05; HR= 0.22 [0.05-1.02]; 95% CI). Similarly; PR negative tumors had a significant higher cumulative probability for progression while moderate and/or strong PR positive tumors linked to lower progressive probability with 68.6 % cumulative PFS at 53 ms (p= 0.02); but no statistical significance difference was found on Cox proportional hazards regression analysis for PFS (p=0.07; HR= 0.22 [0.04-1.14]; 95% CI). Neither ER expression nor PR expression had a significant association with OS (p=0.5, HR= 0.67 [0.21-2.12]; p=0.6, HR= 0.72 [0.91-2.68]; 95% CI, respectively); however, there was a trend for improvement in DFS and PFS favoring moderate/strong ER expression with p-value 0.05 (Table 3, 4) (Figure 2, 3).

**Table 1:** Clinical and Pathological Characteristics

Variable	Summary statistics (percent)
Age/years	
Mean ± SD	53.07±14.19
Median (range)	55 (27-85)
Gender	
Females	14 (46.7)
Males	16 (53.3)
Site	
Anal canal	12 (40)
Colon	7 (23.33)
Rectal	11 (36.67)
Side	
Left	24 (80)
Right	6 (20)
Distance from anal verge	
Mean ± SD	18.97±19.35
Median (range)	10 (2-60)
Grade	
Grade I	6 (20)
Gradde II	18 (60)
Grade III	6 (20)
T Stage	
T2	4 (13.33)
T3	17 (56.67)
T4	9 (30)
N stage	
N0	9 (30)
N1	16 (53)
N2	5 (16.67)
M stage	
M0	19 (63.33)
M1	11 (36.67)
Stage	
Stage I	2 (6.67)
Stage II	7 (23.33)
Stage III	10 (33.33)
Stage IV	11 (36.67)
ER degree	
Negative	12 (40.00)
Weak	11 (36.67)
Moderate	5 (16.67)
Strong	2 (6.67)
PR degree	
Negative	7 (23.33)
Weak	15 (50)
Moderate	7 (23.33)
Strong	1 (3.33)
Chemotherapy lines	
1	9 (30)
2	6 (20)
Variable	Summary statistics (percent)
Chemotherapy lines	
3	12 (40)
4	3 (10)
Response	
Stationary	1 (3.33)
Progressive	21 (70)
Responsive	5 (16.67)
Lost follow up	3 (10)
PFS/month	
Median (range)	9 (4-53)
OS	
Median (range)	27.52 (2.43-58.76)

**Table 2:** Univariate Analysis of Factors Associated with ER/PR Expression

	ER expression			
Variable	Negative (percent)	Positive		*P*-value
		Weak (percent)	Moderate or strong (percent)	
	N=12	N=11	N=7	
T Stage				
T2	1 (8.33)	2 (18.18)	1 (14.29)	0.01*
T3	3 (25)	8 (72.73)	6 (85.71)	
T4	8 (66.67)	1 (9.09)	0	
Response				
Stationary	0	0	1 (16.67)	0.03*
Progressive	10 (100)	9 (81.82)	2 (33.33)	
Responsive	0	2 (18.18)	3 (50)	
T Stage				
T2	1 (14.29)	2 (13.33)	1 (12.5)	0.16
T3	2 (28.57)	8 (53.33)	7 (87.5)	
T4	4 (57.14)	5 (33.33)	0	
Response	N=6	N=14	N=7	
Stationary	0	0	1 (14.29)	0.009*
Progressive	6 (100)	13 (92.86)	2 (28.57)	
Responsive	0	1 (7.14)	4 (57.14)	

*P*-value was calculated by Chi square test; * , Significant P-value

**Table 3:** Cumulative Probability for DFS, PFS and OAS at 12 Months and end of Follow up

	DFS Cumulative survival%		P value	PFS Cumulative survival%		P value	OAS Cumulative survival%		P value
	At 12 months	At last follow up (ms)		At 12 months	At last follow up (ms)		At 12 months	At last follow up (ms)	
ER									
Negative	0.25	0 (at 40ms)	0.06	36.0	0 (at 40ms)	0.01*	100	8.3 (at 58ms)	0.64
Weak	57.1	0 (at 23ms)		18.2	18.2 (at 18ms)		100	28.6 (at 49ms)	
Moderate/strong	75.0	75.0 (53ms)		83.3	66.7 (at 53ms)		100	22.9 (at 52ms)	
PR									
Negative	33.3	0 (at 40ms)	0.12	25.0	0 (at 40ms)	0.02*	100	17.1 (at 58ms)	0.87
Weak	36.4	9.1 (at 26ms)		21.4	7.1 (at 26ms)		100	10.3 (at 49ms)	
Moderate/strong	80.0	80.0 (53ms)		85.7	68.6 (at 53ms)		100	25.0 (at 52ms)	

*P*-value was calculated by the log-rank test; *, Significant P-value

**Table 4:** Cox Proportional Hazards Regression Analysis for DFS, PFS and OAS

	DFS	P value	PFS	P value	OAS	*P*-value
	HR (95% CI)		HR (95% CI)		HR (95% CI)	
ER						
Negative	Ref.		Ref.		Ref.	
Weak	0.53 (0.17-1.69)	0.28	1.72 (0.65-4.60)	0.28	0.64 (0.22-1.87)	0.42
Moderate/strong	0.12 (0.01-1.00)	0.050	0.22 (0.05-1.02)	0.05	0.67 (0.21-2.12)	0.50
PR						
Negative	Ref.		Ref.		Ref.	
Weak	1.00 (0.23-4.38)	0.99	1.36 (0.45-4.04)	0.58	0.92 (0.32-2.66)	0.88
Moderate/strong	0.16 (0.02-1.60)	0.12	0.22 (0.04-1.14)	0.07	0.72 (0.19-2.68)	0.62

*P*-value was calculated by the Hazard ratio

## Discussion

CRC is a major health problem with high mortality rate globally (Haraldsdottir et al., 2014). Sex steroids have been suggested to influence CRC carcinogenesis (Stevanato Filho et al., 2018) and their possible role has been demonstrated in multiple epidemiologic studies (Williams et al., 2016). However, their role as prognostic factors in colon cancer still controversial. This study examined possible correlation of ER/PR expression in CRC with clinical and pathological parameters. 

The current study detected a cytoplasmic expression of ER in 60% of CRC samples. This was in match with results found by Witte et al., (2001) and Xie et al., (2004); they showed nuclear and cytoplasmic immunoreactivity of ER in 67% and 57.5% cases, respectively. These results were slightly different from Rudolph et al., (2012) who found 51.4% positive ER expression rate. In contrary, Slattery et al., (2000) found that all evaluated tumors were ER-negative. 

This difference may be due to differences in the antibody used. Estrogen action is largely mediated by ERα and ERβ, which are members of the nuclear receptor superfamily of transcription factors. The mechanisms underlying the aberrant expression of ER in different human tumors are complex, involving considerable alternative splicing of ERα and ERβ, transcription factors, epigenetic and post-transcriptional regulation of ER expression (Hua et al., 2018). Unlike ERα; which is localized to the nuclei of benign and cancer cells (Xie et al., 2004; Abe et al., 2012), ERβ can be found in nucleus and cytoplasm of normal or cancerous cells. ERβ has at least 5 different isoforms. These isoforms showed different amino acid sequences at the COOH terminus and they are differently expressed in tumor cell lines. Therefore, it is possible that the cytoplasmic immunoreactivity is caused by one of these ER subtypes in CRC (Witte et al., 2001). In our study we used ERβ1 isoform. 

In the current study, PR expression was detected in 76% of cases in contrast to Slattery et al., (2000); who found that only one tumor was PR-positive. This may be due to differences in tissue samples number, technical methods, and the antibody used.

Among different clinical and pathological parameters of the studied CRC cases, a comparative analysis in this study showed significant association between loss of ER expression and depth of invasion (p= 0.01), similar to Wong et al., (2005) who found that lower ER beta expression was associated with higher pT stage and it is in agreement with Hartman et al., (2009); Rudolph et al., (2012) and Ferlay et al., (2012) who found that lack of ER beta expression is associated with advanced cancer stages in CRC. In contrast to this result, Witte et al., (2001) and Xie et al., (2004) didn’t find a significant difference between ER-positive and ER-negative groups with regard to depth of invasion.

In the present study, no significant association was found between ER expression and grades of the investigated tumors and nodal metastasis similar to results reported by Xie et al., (2004), also Witte et al., (2001) didn’t find significant association of ER expression with nodal metastasis. In contrast to Hartman (2009) and Ferlay (2012) who found that the degree of loss of ER beta expression correlated with worsening grade of tumor. In addition to Wong (2005) who found that lower ERβ1 expression was associated with poorer differentiation and higher ERβ2 expression was associated with presence of lymph nodes metastases. This may be due to differences in the antibody used.

In the present study we did not find significant association between PR expression and T stage, grades of the investigated tumors and nodal metastasis. To the best of our knowledge, no previous studies explored this correlation.

This study results showed that stronger ER positivity in CRC sample was associated with a better clinical outcome (p= 0.03). These results were in contrary to Wong (2005) who did not find significant correlation between ERβ protein isoform expression and clinical outcome, this is could be explained by different antibody used and different number of cases in our study. The current results showed similar prognostic significance of PR positivity and clinical behavior in response to treatment (p= 0.009). 

This work showed that the cumulative probability of progression was significantly higher among ER negative population and it was significantly lower in moderate to strong ER positive patients with 66 % cumulative PFS at 53 ms (p= 0.01), however this did not reach statistical significance on Cox proportional hazards regression analysis for PFS (p= 0.05; HR= 0.22, [0.05-1.02]; 95% CI), these results are in agreement with Rudolph et al., (2012) who found that ER beta negativity was associated with an increased hazard ratio for death (P=0.02; HR=1.61, [1.09-2.40]; 95% CI), and death attributed to CRC (P=0.06; HR=1.54, [0.99-2.39]; 95% CI) as well as a poorer DFS (DFS P=0.04; HR=1.64, [1.23-3.36]; 95% CI). But Witte (2001) did not find a significant difference between the ER positive and ER negative groups with OS. Similarly, the study found that PR negative tumors had highest probability to progress while it is significantly lower in moderate to strong PR positive patients with 68.6% cumulative PFS at 53 ms (p= 0.02), but this did not prove true or statistically significant on Cox proportional hazards regression analysis for PFS (p=0.07; HR= 0.22 [0.04-1.14]; 95% CI) (Tab. 3, 4) (Figure 2, 3).

Neither ER expression nor PR expression had a significant association with OS (p=0.5, HR= 0.67 [0.21-2.12]; p=0.6, HR= 0.72 [0.91-2.68]; 95% CI, respectively); however, there was a trend for improvement in DFS and PFS favoring moderate/strong ER expression with p-value 0.05.

## Limitations

Small sample size makes it difficult to extrapolate population based results.

In conclusion, this study showed that ER expression on CRC samples was associated with smaller tumor size; also, ER/PR expression associated with better prognosis, lower progression and better clinical outcome. This could be the bases for exploring possible therapeutic applications of Estrogens, progestins, ER and PR against CRC. Analysis of hormonal status in CRC may be of clinical importance. Further confirmatory studies among larger number of cases are necessary.

## References

[B1] Abe K, Miki Y, Ono K (2010). Highly concordant coexpression of aromatase and estrogen receptor beta in non-small cell lung cancer. Hum Pathol.

[B3] Ascenzi P, Bocedi A, Marino M (2006). Structure-function relationship of estrogen receptor alpha and beta: impact on human health. Mol Aspects Med.

[B4] Bray F, Ferlay J, Soerjomataram I (2018). Global cancer statistics 2018: GLOBOCAN estimates of incidence and mortality worldwide for 36 cancers in 185 countries. CA Cancer J Clin.

[B5] Burns KA, Korach KS (2012). Estrogen receptors and human disease: an update. Arch Toxicol.

[B6] Campbell-Thompson M, Lynch IJ, Bhardwaj B (2001). Expression of estrogen receptor (ER) subtypes and ERbeta isoforms in colon cancer. Cancer Res.

[B7] Deroo BJ, Korach KS (2006). Estrogen receptors and human disease. J Clin Invest.

[B8] Ferlay J, Soerjomataram I, DikshitR (2014). Cancer incidence and mortality worldwide: sources, methods and major patterns in GLOBOCAN 2012. Int J Cancer.

[B9] Foley EF, Jazaeri AA, Shupnik MA, Jazaeri O, Rice LW (2000). Selective loss of estrogen receptor beta in malignant human colon. Cancer Res.

[B10] Fong D, Seeber A, Terracciano L (2014). Expression of EpCAMMF and EpCAMMT variants in human carcinomas. J Clin Pathol.

[B11] Foster PA (2013). Oestrogen and colorectal cancer: mechanisms and controversies. Int J Colorectal Dis.

[B12] Haraldsdottir S, Einarsdottir HM, Smaradottir A, Gunnlaugsson A, Halfdanarson TR (2014). Colorectal cancer-review. Laeknabladid.

[B13] Härkönen PL, Mäkelä SI (2004). Role of estrogens in development of prostate cancer. J Steroid Biochem Mol Biol.

[B14] Hartman J, Edvardsson K, Lindberg K (2009). Tumor repressive functions of estrogen receptor beta in SW480 colon cancer cells. Cancer Res.

[B15] Heijmans J, Muncan V, Jacobs RJ (2011). Intestinal tumorigenesis is not affected by progesterone signaling in rodent models. PLoS One.

[B16] Hogan AM, Collins D, Baird AW, Winter DC (2009). Estrogen and gastrointestinal malignancy. Mol Cell Endocrinol.

[B17] Hua H, Zhang H, Kong Q, Jiang Y (2018). Mechanisms for estrogen receptor expression in human cancer. Exp Hematol Oncol.

[B18] Ibrahim AS, Khaled HM, Mikhail NN, Baraka H, Kamel H (2014). Cancer incidence in Egypt: results of the national population-based cancer registry program. J Cancer Epidemiol.

[B19] Jordan VC (2007). Chemoprevention of breast cancer with selective oestrogen- receptor modulators. Nat Rev Cancer.

[B20] Konstantinopoulos PA, Kominea A, Vandoros G (2003). Oestrogen receptor beta (ERβ) is abundantly expressed in normal colonic mucosa, but declines in colon adenocarcinoma paralleling the tumour’s dedifferentiation. Eur J Cancer.

[B21] Qasim BJ, Ali HH, Hussein AG (2011). Immunohistochemical expression of estrogen and progesterone receptors in human colorectal adenoma and carcinoma using specified automated cellular image analysis system: A clinicopathological study. Oman Med J.

[B22] Rossouw JE, Anderson GL, Prentice RL (2002). Risks and benefits of estrogen plus progestin in healthy postmenopausal women: principal results From the Women’s Health Initiative randomized controlled trial. JAMA.

[B23] Rudolph A, Toth C, Hoffmeister M (2012). Expression of oestrogen receptor b and prognosis of colorectal cancer. Br J Cancer.

[B24] Sasso CV, Santiano FE, Verde Arboccó FC (2019). Estradiol and progesterone regulate proliferation and apoptosis in colon cancer. Endocr Connect.

[B25] Siegfried JM (2014). Smoking out reproductive hormone actions in lung cancer. Mol Cancer Res.

[B26] Slattery ML, Samowitz WS, Holden JA (2000). Estrogen and progesterone receptors in colon tumors. Am J Clin Pathol.

[B27] Stevanato Filho PR, Aguiar Júnior S, Begnami MDMD (2018). Estrogen receptor β as a prognostic marker of tumor progression in colorectal cancer with familial adenomatous polyposis and sporadic polyps. Pathol Oncol Res.

[B28] Syed V, Ulinski G, Mok SC, Yiu GK, HoS-M (2001). Expression of gonado tropin receptor and growth responses to key reproductive hormones in normal and malignant human ovarian surface epithelial cells. Cancer Res.

[B29] Topi G, Ehrnström R, Jirström K (2017). Association of the oestrogen receptor beta with hormone status and prognosis in a cohort of female patients with colorectal cancer. Eur J Cancer.

[B30] Wada-Hiraike O, Imamov O, Hiraike H (2006). Role of estrogen receptor beta in colonic epithelium. Proc Natl Acad Sci U S A.

[B31] Weyant MJ, Carothers AM, Mahmoud NN (2001). Reciprocal expression of ERalpha and ERbeta is associated with estrogen-mediated modulation of intestinal tumorigenesis. Cancer Res.

[B32] Williams C, DiLeo A, Niv Y, Gustafsson JA (2016). Estrogen receptor Beta as target for colorectal cancer prevention. Cancer Lett.

[B33] Witte D, Chirala M, Younes A, Li Y, Younes M (2001). Estrogen receptor is expressed in human colorectal adenocarcinoma. Hum Pathol.

[B34] Wong N, Malcomson RDG, Jodrell DI (2005). ERbeta isoform expression in colorectal carcinoma: an in vivo and in vitro study of clinicopathological and molecular correlates. J Pathol.

[B35] Xie LQ, Yu JP, Luo HS (2004). Expression of estrogen receptor β in human colorectal cancer. World J Gastroenterol.

[B36] Zannoni GF, Monterossi G, De Stefano I (2013). The expression ratios of estrogen receptor a (ERa) to estrogen receptor b1 (ERb1) and ERa to ERb2 identify poor clinical outcome in endometrioid endometrial cancer. Hum Pathol.

